# Using incident reports to diagnose communication challenges for precision intervention in learning health systems: A methods paper

**DOI:** 10.1002/lrh2.10425

**Published:** 2024-05-09

**Authors:** Rebecca R. S. Clark, Tamar Klaiman, Kathy Sliwinski, Rebecca F. Hamm, Emilia Flores

**Affiliations:** ^1^ Center for Health Outcomes and Policy Research University of Pennsylvania School of Nursing Philadelphia Pennsylvania USA; ^2^ University of Pennsylvania Health System Philadelphia Pennsylvania USA; ^3^ Leonard Davis Institute of Health Economics Philadelphia Pennsylvania USA; ^4^ University of Pennsylvania Perelman School of Medicine Philadelphia Pennsylvania USA; ^5^ Division of Maternal‐Fetal Medicine University of Pennsylvania Perelman School of Medicine Philadelphia Pennsylvania USA; ^6^ Center for Evidence‐Based Practice University of Pennsylvania Health System Philadelphia Pennsylvania USA

**Keywords:** communication, health equity, healthcare team, incident reports, maternal health

## Abstract

**Introduction:**

Poor communication is a leading root cause of preventable maternal mortality in the United States. Communication challenges are compounded with the presence of biases, including racism. Hospital administrators and clinicians are often aware that communication is a problem, but understanding where to intervene can be difficult to determine. While clinical leadership routinely reviews incident reports and acts on them to improve care, we hypothesized that reviewing incident reports in a systematic way might reveal thematic patterns, providing targeted opportunities to improve communication in direct interaction with patients and within the healthcare team itself.

**Methods:**

We abstracted incident reports from the Women's Health service and linked them with patient charts to join patient's race/ethnicity, birth outcome, and presence of maternal morbidity and mortality to the incident report. We conducted a qualitative content analysis of incident reports using an inductive and deductive approach to categorizing communication challenges. We then described the intersection of different types of communication challenges with patient race/ethnicity and morbidity outcomes.

**Results:**

The use of incident reports to conduct research on communication was new for the health system. Conversations with health system‐level stakeholders were important to determine the best way to manage data. We developed a thematic codebook based on prior research in healthcare communication. We found that we needed to add codes that were equity focused, as this was missing from the existing codebook. We also found that clinical and contextual expertise was necessary for conducting the analysis—requiring more resources to conduct coding than initially estimated. We shared our findings back with leadership iteratively during the work.

**Conclusions:**

Incident reports represent a promising source of health system data for rapid improvement to transform organizational practice around communication. There are barriers to conducting this work in a rapid manner, however, that require further iteration and innovation.

## 1 INTRODUCTION

The United States has the worst severe maternal morbidity (SMM) and mortality rates of any high‐income country, and it is driven by a persistent, widening racial disparity. SMM and mortality in the United States is a burden persistently and disproportionately borne by Black women.^1^ In 2020, the Black maternal mortality rate was almost three times higher than that for White women and these have increased significantly from previous years, whereas rates for white women have remained flat.^1^, ^2^, ^3^

Communication is a leading root cause of preventable maternal mortality and morbidity.^4^ The Agency for Healthcare Research and Quality issued a report on the state of the science on the contributions of diagnostic errors on SMM^5^ and identified poor communication, including delays in assessing clinical warning signs, as a major contributor to adverse outcomes. Delays in assessing warning signs can occur for many reasons, but medical gaslighting, in which members of the healthcare team dismiss or deny a patient's, or another member of the team's, understanding or knowledge, and which may have racist underpinnings, is one way that might occur.^6^, ^7^ A significant body of literature indicates that communication between clinicians and Black women is a significant problem, with clinicians not listening to Black women or communicating in a respectful, person‐centered manner.^8^, ^9^, ^10^, ^11^, ^12^, ^13^, ^14^, ^15^ Research by Peek et al. has shown that prejudice and discrimination manifest in many ways, including disrespect and failure to communicate options.^16^ Dahm et al. have also shown associations between common cognitive biases and communication challenges but have taken this research further to also show the association between diagnostic errors and patient safety.^17^ These researchers have shown how labeling bias (e.g., conversations within the healthcare team about a patient being a “frequent flyer” or “drug seeking”) can affect or negatively influence how information is interpreted by the rest of the healthcare team and result in less responsiveness to patient complaints.^17^ There is also evidence to suggest that communication within the healthcare team varies when caring for a Black patient.^18^ Professional organizations and national stakeholders have developed guidelines to address poor communication; however, gaps remain for Black women in the maternity setting in the United States.^19^, ^20^

A recent systematic review of implementation science methods in maternity care found many studies that identified effective communication as a critical component to improving maternity outcomes (*n* = 78/158).^21^ To say that “communication” is the issue is paralyzing because it is so vast and encompasses so much. How can healthcare settings identify which specific communication challenges they are dealing with, and comprehend the nature of these challenges, to appropriately intervene and improve healthcare quality, safety, and outcomes? Further, how can learning health systems leverage data that already exists, as opposed to generating new data, to do this work? We were inspired by a research study that used incident reports to understand communication challenges in a medical setting.^22^ Incident reporting systems are required by the Joint Commission; therefore, incident reports exist in every hospital. Specifically, incident reporting systems are typically electronic, separate from the electronic health record (EHR), and exist to identify and elevate safety concerns to management. These reports, therefore, vary from surgical instruments that are not appropriately clean, to emergency pagers not working to unprofessional interactions between healthcare team members and poor patient outcomes. Reporting is not mandated, so what gets reported may vary for many reasons—particular incidents that a service line wants to track, a healthcare team member's awareness of something impacting care or being reportable, having the time to file a report, or a team member's perceptions of a hospital's safety culture (i.e., being able to report without personal reprisal). These reports may (not) be placed anonymously. These reports are typically reviewed on a daily basis by pertinent clinical leaders, with actions being taken as needed to address the issues raised in the clinical reports. Incident reports are unique in being (1) voluntarily created by hospital staff, (2) potentially anonymous, and (3) linkable with the EHR. As such, incident reports provide a perspective on the working of the healthcare team and the organization that are not as readily captured by patient grievances or post‐hospitalization surveys such as the Hospital Consumer Assessment of Healthcare Providers and Systems or Press‐Ganey. Further, due to the ability to link incident reports with the EHR, there is an ability to assess variation and disparities across patient populations or units. In other words, incident reports are, par excellence, the kind of data that learning health systems can leverage for continuous improvement.^23^ The difficulty, of course, is in figuring out how best to leverage the rich data captured by incident reports to support continuous process improvement.^24^ To address communication as a leading cause of preventable maternal morbidity and mortality, we need to be able to precisely understand the challenges we are facing, and how they are associated with poor patient outcomes, to motivate and inform continuous improvement and innovation in the healthcare setting.

## 2 QUESTION(S) OF INTEREST OR RESEARCH INTERESTS

Accordingly, our research interest was to use incident reports to understand communication challenges and opportunities, especially with regard to disparate care and outcomes, in the Women's Health service in a large, urban hospital in an academic medical system. We hypothesized that incident reports would allow us to precisely identify communication challenges that could be addressed to improve care quality, safety, and outcomes, as well as team functioning. Our purpose in this paper is to share our process of and learnings from using a non‐EHR‐based data source to generate evidence and learnings through data collected from service delivery to identify opportunities for improvement and innovation in communication and equity therein.

## 3 METHODS

### 3.1 Study design and sample

We conducted a qualitative content analysis of incident reports using a constant comparative method and an inductive and deductive approach to understand communication challenges. Next, we evaluated how these communication challenges were associated with patient race/ethnicity and SMM. We analyzed incident reports between 2019 and 2022 which were written and submitted electronically by hospital staff from the maternity units (antepartum, labor and birth, postpartum) of a large, urban, community hospital in the mid‐Atlantic region, with over 5000 births per year (a little under 30% of those births being to Black women). All fields in the incident report were filled out by the filing staff member. The one exception to this was the assessment of injury severity, which was filled out or updated by the hospital's safety personnel. This study was deemed exempt by the institution's Institutional Review Board, as the data analyzed did not contain identifiable information.

### 3.2 Data governance

We planned that we would access the data in a similar manner to how other health system data is accessed, and that it was governed in a similar manner. We had initially conceptualized that the data were governed at the hospital level, as many projects using hospital data do not require health system‐level conversations. This is, in part, due to the pre‐existence of policies around work using hospital data in a learning health system. We found that, due to the novel nature of using incident reports, there was not (at the time) an established protocol for the use of the data. As a result, we had conversations with leadership at the corporate level of the health system (instead of staying at the hospital level) to discuss issues of process, privacy, and use. A plan was made in consultation with the Chief Privacy Officer and the Executive Director of Clinical Research Operations to work with the Chief Research Information Officer's office for the deidentification of the incident reports using PHIlter.^25^, ^26^ A further part of this plan was to have a secure research drive created within the health system's computing infrastructure so the incident reports, though deidentified, would not leave the health system. The initial plan had been to store and analyze the incident reports in a secure research drive within the university (of which the health system is a part). The decision to create a secure research drive within the health system highlights the potential unique security needs around incident reports. The process also highlights the lack of frequency with which incident reports are used in this manner and an opportunity to create protocols to support further learnings for the health system. As these things were accomplished, we received approval to conduct the study from our institutional review board.

### 3.3 Data acquisition and management

We collaborated with the quality improvement (QI) specialist assigned to the Women's Health units to identify and extract all incident reports from the antepartum, labor and birth, and postpartum units that were submitted between 2019 and 2022. Incident report categories that were unlikely to include communication as a root cause were excluded. The QI specialist linked the incident reports to the patient involved via the medical record number where possible (not all incident reports involved a patient). Where the linkage was possible, the QI specialist linked the patient's race, ethnicity, birth outcome, and morbidity outcomes to the incident report. The QI specialist then shared this data via secure share with an analyst in the health system's Chief Information Officer's office. The analyst ran the data through PHIlter^25^, ^26^ to deidentify data. We worked iteratively with the analyst to fine‐tune the algorithm, for example, adding proper names of obstetric maneuvers or medications so that they would not be redacted. The deidentified data were saved in a secure drive established for the research team to ensure that all the data remained in the health system IT infrastructure.

The analyst working with PHIlter initially sent us 1006 incident reports. We began to identify the sample from this data. A code error resulted in roughly an additional 2324 incident reports for inclusion in the dataset. The team decided to continue with the initial dataset we had received and to hold the additional 2324 reports in reserve for a few reasons: (1) the first set of incident reports was randomly drawn, spanning the entire time period; (2) we had already begun sample identification and to add the additional 2324 would have required starting over with an unfeasibly large sample and; (3) the team determined that the additional incident reports could be used for testing and validating an algorithm to support health systems in learning from their incident report data. The research team stored the data in a cloud‐based, HIPAA‐compliant service, and used Atlas.ti^27^—a cloud‐based qualitative analytic software that received health system approval—to conduct the qualitative analysis.

### 3.4 Sample identification

We had two team members (one with clinical expertise) review the incident reports independently and determine the incident report's eligibility for sample inclusion. Inclusion criteria were whether the incident report described a communication issue (positive or negative).^22^ The communication challenge could be between the healthcare team or between the healthcare team and the patient. All patient demographic data were blinded until all data were coded. We found that clinical expertise was necessary to understand the incident reports, so we reviewed the incident reports independently for inclusion, and the team members who did not have clinical expertise marked incident reports that required clinical explanation to determine inclusion. The two team members met weekly to review differences in inclusion and resolve discrepancies and brought the remaining questions or discrepancies to the full team weekly meeting. Of the 1006 incident reports, we identified 542 as representing communication challenges.

### 3.5 Coding and analysis

Following the sample identification, we used a pre‐existing codebook from the work of Umberfield et al.^22^ This codebook used Lingard et al.'s framework^28^ as expanded by Halverson et al.^29^ To this we added codes from Singh and Sittig^30^ and also allowed for *inductive* codes that arose from the data. Please see Appendix 1 for the codebook. At least three members of the research team met weekly to review incident reports and code the relevant communication failures.

One of these team members was a qualitative research expert and the other was a registered nurse (though without significant maternity care experience). These team members found that they had significant disagreements about the coding. We determined that maternity clinical expertise was necessary to understand what was occurring in the incident report. Contextual expertise and interdisciplinary perspectives were critical for the work and present within the larger team. For instance, knowing what policies were in place, what policy changes were occurring, or understanding dynamics in how the units run is crucial to the work, as is having perspectives from multiple disciplines. The team decided to redo the coding with the addition of another team member who was part of the sample creation duo and had pertinent clinical expertise. We proceeded to conduct a qualitative content analysis using a constant comparative method in which the three team members reviewed each report, identified communication challenge types, and applied corresponding codes. We used memos, an audit trail of deliberations and decisions, and weekly meetings with the wider team to resolve discrepancies, achieve consensus, and increase rigor. After completing the initial coding, we reviewed codes for consistency across incident reports. We found that the *inductive* codes that arose from the data mostly centered on issues of equity in communication, as such issues were not covered by the existing codebook.

We shared our ongoing process with stakeholder leadership, who were very interested in and supportive of our work and findings which were using historic incident reports. We were also asked to share the ongoing work with clinical work groups invested in ongoing quality and patient care improvement. This was an important part of the process because it created a dialogue and allowed for a flow of important information, such as clinical policy change or ongoing initiatives, between the clinical leadership and the research team. Apart from the one team member who is also part of the women's health QI committee, stakeholder leadership was not involved in this research. Clinical leadership, of course, continued their standard practice of daily incident report review and process improvement.

## 4 RESULTS

We learned several lessons from this process: (1) unique data governance needs, including appropriate health system and hospital leadership stakeholder involvement; (2) the challenges of using natural language processing (NLP) algorithms for deidentifying data; (3) the critical importance of clinical and contextual expertise on the team for this work; (4) the difficulty of using incident reports with regards to the effort and time needed to analyze them; and (5) the importance of leveraging incident reports by reviewing batches of them over time to understand larger themes or deeper aspects of context‐specific opportunities for improvement.

We used incident reports in a novel way and there was no pre‐existing data governance structure in place for doing so (i.e., whose permission is necessary to use the data, how to gain that permission, how to use and store the data, etc.). This is in contrast to EHR data, for example, for which clear data governance structures were already in place. In a learning health system, where a wide array of data is available for analysis to support clinical and operational work, it is important to engage with stakeholders when data is being used in a novel manner. Incident reports, in and of themselves, are common in healthcare settings. To use them for research, and to use a large sample of them to develop both a thematic understanding of communication issues and to pinpoint issues for intervention, is not common. It is not always easy to identify the pertinent stakeholders in a complex health system. Asking who the stakeholders are, and continuing to ask, if necessary, is important for data governance due process and for the work to have its best effect.

NLP algorithms, like PHIlter, come with costs and benefits. These algorithms can make deidentifying data much faster. In our case, we considered having the principal investigator deidentify the incident reports by hand. This would have involved significant time and effort. By collaborating with our QI specialist and the research informatics analyst, we were also able to decrease the number of people with potential access to identifiable data and to set up a process where the data was deidentified in a more secure manner. We were fortunate to be able to work with an experienced analyst who was able to run PHIlter on our behalf. The presence of an analyst with this kind of expertise, in and of itself, is a mark of the importance the learning health system places on this kind of work and the resources available in the wider system. The main challenge posed by using an NLP algorithm is that it required “training”—adding the proper names of obstetric instruments or procedures, for example, so that they would not be redacted. Further, since this deidentification process was being done on our behalf due to data security and governance concerns, we did not have a way to check the algorithm code. The mistake in code that resulted in a second sample that was three times larger than the first led to two learnings for future work: (1) find a way to have the code checked prior to starting on sample creation and (2) consider further restricting the time period from which incident reports are drawn since over 3000 incident reports were larger than needed for this initial qualitative work.

The process of conducting this work was an object lesson in the necessity of having clinical, contextual, interprofessional, and methodologic expertise on the team. The full team included people with nursing, midwifery, maternal‐fetal medicine, qualitative, and context‐specific expertise. Some people represented multiple areas of expertise. We found that, when we broke into smaller teams to determine sample inclusion or analyze the data, maternity‐specific clinical expertise was a necessary requirement for one of the team members. Specifically, we had initially thought two members of the research team representing research and general nursing expertise would be sufficient for determining what kind of communication issues were present in individual incident reports. The team member with nursing expertise had completed a maternity rotation and clinical, but it was not an area of expertise. We found, however, that a team member with maternity‐specific clinical expertise was needed to explain the clinical content of the incident report and that this was necessary to understand the communication challenges present. Context‐specific knowledge, that is, familiarity with the specific units represented in the incident reports, was important to have, but it was sufficient to have it present on the larger team. Methodologic expertise was critical for conducting analyses. Having multiple professionals present on the team was important for larger conversations around interpreting the incident reports (e.g., team members with different professional backgrounds understood the incident reports differently and interpretation was assisted by having interdisciplinary conversations). If other learning health systems were interested in conducting similar work, these would be important considerations for determining who was on the team.

Our initial approach to the analysis of incident reports was time and effort intensive. This is appropriate for methodologically robust qualitative research. The tension between the need for speed and rigor in research to support learning health systems' improvement processes in real time is well documented in the literature.^31^ One consideration for others interested in trying this process would be to use a rapid thematic analysis approach.^32^ Even rapid thematic analysis, however, is time intensive and can take long periods of time if the team has only small amounts of effort to dedicate to the project.^33^

The above three points showcase the significant resources—effort, time and expertise—needed to use incident reports as depicted. This same process, however, affords a critical opportunity for learning health systems to precisely identify and understand context‐specific opportunities for improvement. This same opportunity is not afforded by routine daily or weekly reviews of incident reports, even though that process is also important. It remains to be seen if there are ways to make this process easier and faster.

The main opportunities for this protocol and its potential impact are trifold: (1) informing the hospital clinicians and leadership of specific targets to intervene to improve healthcare team and team‐patient communication to improve care and outcomes; (2) providing guidance to other health systems and hospitals about a method to leverage their incident reports to understand their particular needs for improvement; and (3) developing understanding about the specific ways in which communication is a root cause of preventable maternal morbidity and mortality and generating hypotheses about ways to address these specific ways. Hospital clinicians and leadership in our learning health system are aware that communication is an issue, and review incident reports on a regular basis to improve care and outcomes. Using incident reports in the manner described above, however, allows for the identification of what specific aspects of communication need to be addressed to improve team functioning, patient care, and outcomes. The systematic approach outlined in the protocol allows us to take a broader view, identifying themes and trends in communication challenges and how these are associated with morbidity outcomes and disparities therein.

Incident reports are a rich source of data. Publishing this protocol provides a template for others in learning health systems who want to use their hospital or health system's data to perform “precision improvement” to improve care quality and outcomes. Ultimately, we also hope that the impact of this work will include a deeper understanding of how communication challenges are associated with patient harm and racially disparate birth and morbidity outcomes so that we can begin to understand where to direct our interventions to improve communication in inpatient maternity care.

## 5 DISCUSSION

In this paper, we presented our method of using incident reports, a non‐EHR based data source, to understand our context‐specific communication challenges in the Women's Health service to inform and share tactical recommendations/opportunities for improvement in communication. Incident reports present an important data source to be leveraged to pinpoint specific issues to improve communication or other aspects of clinical care, including a potential way of identifying issues related to improving equity in care and outcomes. We found that this process, while rich and rewarding, also requires specialized knowledge (context, clinical, professional, and research) represented in the team. The novel contribution of this work is how we used incident reports in conjunction with data from the EHR to identify opportunities to address potential disparities in communication.

We want to note two challenges to this method. The first challenge is that the healthcare setting is dynamic, constantly changing in efforts to improve care and outcomes. Some of the specific communication challenge items may have already been noted and addressed by clinicians and leadership. We think that conducting this work, however, is an opportunity to step back and reflect on changes that have been made, whether they have been successful or sustained, and where effort might need to be redirected. We also have input and representation from two of the major disciplines involved with care in the inpatient maternity setting, nursing, and medicine. A second challenge in this research is that the resources involved in leveraging a hospital's data in this way are significant, including time, effort, and expertise (qualitative, clinical, etc.). This becomes a challenge to recommending how other institutions might conduct this work. One possibility is to create a tool that would allow clinical leaders to keep track of the “big picture” being painted by incident reports over time while also maintaining daily reviews of incident reports. Developing such a tool, or tools, in future research would create the potential to do this work in less resource‐intensive ways that would support the continuous process improvement work of learning health systems.

Using incident reports is not without limitations. Communication is complex, with multiple parties involved, and incident reports represent a single perspective. As such, individual incident reports are limited as to the insight they provide regarding ongoing communication concerns (or any ongoing quality issue) in a learning health system. Taken en masse, however, we believe that they have the capacity to illuminate targets for QI intervention. Incident reports are also not mandatory, and healthcare team members may not submit them after critical incidents for a wide array of reasons. Again, this underlines the importance of looking at a body of incident reports to understand larger trends and needs in the health system.

## 6 CONCLUSION

Incident reports represent a promising source of health system data for informing system evaluations designed to achieve continuous rapid improvement in health and healthcare and to transform organizational practice and increase equity. There are barriers to conducting this work in a rapid manner, however, that require further iteration and innovation.

## CONFLICT OF INTEREST STATEMENT

The authors declare no conflicts of interest.

## Supporting information


**Data S1.** Supporting Information.
