# Supplementary material for: Using incident reports to diagnose communication challenges for precision intervention in learning health systems: A methods paper
Source: Learn Health Syst. 2024 May 9;8(Suppl 1):e10425. doi: 10.1002/lrh2.10425 (PMC11176586; doi:10.1002/lrh2.10425)
Supplement: Supplementary file 1 — Data S1. Supporting Information. [file LRH2-8-e10425-s001.docx]

| Appendix 1: Codebook | |
| --- | --- |
| Code | Meaning |
| Contextual Failures | |
| Audience | Refers to the participants present during an exchange; gaps in the composition of the group engaged in the communication  Reflected situations in which a communicative exchange excluded a key person  Appropriate individuals were not participating |
| Content | Content consisted of communicative exchanges that contained incomplete or inaccurate information  Information in the message was inaccurate, missing, or unclear |
| Omission | Necessary communication was absent |
| Occasion | Refers to the physical and temporal situation of an exchange; Problems in the situation or context of the communication event  Occasion included problems related to time and space. |
| Purpose | Refers to the goals, implicit or explicit, of the communication; Communication events in which purpose is unclear, not achieved, or inappropriate  Included situations in which questions were asked by one team member, but not responded to by the team, prompting repeated and increasingly urgent requests.  Also includes patient requests being overlooked or ignored |
| Inappropriate | Offensive remarks (in exchange or in incident report) or unreasonable requests (can be coded with more than one contextual failure) |
| Failure Type^23,24^ | |
| Transfer of Information | When the information exchange between communicators was ineffective or insufficient; poor or unsuccessful information exchange |
| Lack of Shared Understanding | Shared understanding occurs when communication integrates multiple perspectives and gets communicators on the same page. A lack of shared understanding occurs when the communication is ineffective or insufficient in integrating the perspectives of those involved in the communication; failure to integrate multiple perspectives.  Two parties are not aligning in their understanding of something |
| Sociotechnical Dimensions^25^ | |
| Clinical Content | The text, numeric data and images that constitute the ‘language’ of clinical applications |
| Hardware and Software | Computing infrastructure used to support and operate clinical applications and devices |
| Human-computer interface | All aspects of technology that users can see, touch or hear as they interact with it |
| Internal organizational features | Policies, procedures, work environment and culture  Policy not followed or lack of policy |
| Workflow and communication | Processes to ensure that patient care is carried out effectively |
| People | Everyone who is involved with patient care and/or interacts in some way with healthcare delivery (including technology). This would include patients, hospital personnel that are not direct caregivers, information technology (IT) developers and other IT personnel and informaticians |
| Inductive Codes | |
| Against Medical Advice | Against medical advice; penalization ("non compliant") |
| Authority | When an authority figure is called to deal with a patient issue (security, management, etc.) OR when authority should have been called and was not. |
| Blame Shifting | Placing blame on others |
| Language Translation | Issues around language interpretation |
| Technical | We are defining this as related to technology (i.e., omnicell) |
| Prescription Release | When wrong order set gets released |
| Positive Communication | |
| Positive Communication | Examples of effective communication |
